# Ultrastructural alterations in *Plasmodium falciparum* induced by chalcone derivatives

**DOI:** 10.1186/s13104-020-05132-z

**Published:** 2020-06-15

**Authors:** Shweta Sinha, B. D. Radotra, Bikash Medhi, Daniela I. Batovska, Nadezhda Markova, Rakesh Sehgal

**Affiliations:** 1 Department of Medical Parasitology, Post Graduate Institute of Medical Education and Research, Chandigarh, 160012 India; 2 Department of Histopathology, Post Graduate Institute of Medical Education and Research, Chandigarh, India; 3 Department of Pharmacology, Post Graduate Institute of Medical Education and Research, Chandigarh, India; 4 Institute of Organic Chemistry with Centre of Phytochemistry, Bulgarian Academy of Sciences, Sofia, Bulgaria

**Keywords:** *Plasmodium falciparum*, Transmission electron microscopy, Chalcones, Malaria, In vitro

## Abstract

**Objective:**

Chalcones (1, 3-diaryl-2-propen-1-ones) and their derivatives are widely explored from the past decade for its antimalarial activity. To elucidate their mechanism of action on the malaria parasite, the ultrastructural changes with the action of these derivatives in different organelles of the parasite were studied in vitro. Infected RBCs [CQ sensitive (MRC-2) and CQ resistant (RKL-9) *Plasmodium* strain] were treated with three chalcone derivatives 1, 2 and 3 and standard drugs, i.e., CQ and artemisinin at twice their respective IC_50_ values for 24 h and then harvested, washed, fixed, embedded and stained to visualize ultra-structure changes before and after intervention of treatment under in vitro condition through transmission electron microscope.

**Results:**

The ultrastructural changes demonstrate the significant disturbance of all parasite membranes, including those of the nucleus, mitochondria and food vacuole, in association with a marked reduction of ribosomes in the trophozoites and cessation of developing schizonts which suggest multiple mechanisms of action by which chalcone derivatives act on the malaria parasite. The present study opens up perspectives for further exploration of these derivatives in vivo malaria model to discover more about its effect and mechanism of action.

## Introduction

Malaria caused by *Plasmodium falciparum* species is the most noxious as they can infect all RBCs irrespective of their ages. The species is also most prevalent in the WHO African Region, leading to almost 99.7% malaria cases in 2018 [1]. Although there is a significant reduction in the number of malaria-infected cases and deaths over years of successful efforts through the malaria elimination programme, at the same time with the persistence evidence of growing *P. falciparum* resistance to artemisinin has led to a global threat [2–5]. Therefore, it is crucial to discover some fresh antimalarial drug entities which have the quality of effectiveness as well as efficiency towards malaria treatment [6] and can counter the rapidity of the drug resistance phenomenon of the parasite.

Chalcones (1,3-diaryl-2-propen-1-ones), a plant secondary metabolites is well known for its diverse pharmacological activity [7–9], including antimalarial activity [10]. It can also offer a huge repository of bioactive compounds with enormous molecular targets [11]. Earlier our group has reported three potent chalcone derivatives 1, 2 and 3 with antimalarial activity, screened from a series of recently synthesized chalcone derivatives under in vitro conditions on both chloroquine-sensitive and chloroquine-resistant strains of *Plasmodium*. The study revealed even minor structural changes can increase the activity of a particular pharmacophore. Additionally, these derivatives act on the haeme degradation pathway of the malaria parasite, i.e., in a similar way as chloroquine does [11]. However, to better understand the effect and the mechanism by which these derivatives act on *P. falciparum*, an ultrastructure study was performed using the in vitro culture system.

## Main text

### Materials and methods

#### Parasites

The CQ-sensitive (MRC-2) and CQ-resistant (RKL-9) *P. falciparum* strains were maintained aseptically in continuous culture as mentioned earlier [12]. The *P. falciparum* culture was nurtured in A+ erythrocytes at 5% haematocrit. The complete culture medium consisted of RPMI 1640 (with glutamine, but without NaHCO_3_), constituted of 5.94 g/L of HEPES buffer, 1.00 g/L dextrose, 40.00 mg/L of gentamycin. In addition, supplemented with 5% NaHCO_3_ and 10% (v/v) inactivated human AB+ serum. Parasite cultures were kept at 37 °C with 90% N_2_, 5% O_2_ and 5% CO_2_, and the culture medium was changed at the interval of 22–24 h. Parasites were synchronized to the ring stage using 5% d-sorbitol as described previously [13]. Parasite growth and multiplication were checked by thin smear Giemsa stained slides using a light microscope.

#### Drugs and drug exposure

The three chalcone derivatives 1, 2 and 3 were synthesized as described previously by our group [11]. Chloroquine phosphate was obtained from Sigma Aldrich and artemisinin from IPCA. Stock solutions of three chalcone derivatives, chloroquine phosphate and artemisinin were separately prepared by dissolving each one of them in the diluted concentration of DMSO (1%) to attain a concentration of 1.00 mg/mL.

Before each experiment, cultures were expanded in sterile cell culture plates, maintaining less than 5% parasitaemia in 5% haematocrit. A non-treated negative control was maintained separately in a different plate at the same culture condition, i.e., at 37 °C, 5% CO2, 90% N_2_, 5% O_2_. The ring-stage parasite-infected RBCs (strain MRC-2 & RKL-9) were exposed to different drugs for 24 h at twice their respective IC_50_ values given in Table 1. After drug exposure, the iRBC were washed and lysed with 1X RBC lysis buffer to release parasite, which was further processed for transmission electron microscopy to assess parasite ultrastructure.

**Table 1:** IC_50_ value of chalcone derivatives and standard drugs [11]

Drugs/compound name	Structure	Series name	MRC-2 (*P. falciparum* CQ^S^ strain) IC50 (µg/mL)	RKL-9 (*P. falciparum* CQ^R^ strain) IC50 (µg/mL)
(*E*)-1-(2,5-Dimethoxyphenyl)-3-(4-methoxyphenyl)prop-2-en-1-one		1	O.13	0.14
(*E*)-(3,4,5-Trimethoxyphenyl)-3-(4-methoxyphenyl)prop-2-en-1-one		2	0.35	0.19
(*E*)-1-(3,4,5-Trimethoxyphenyl)-3-(3,4-dimethoxyphenyl)prop-2-en-1-one		3	0.11	0.18
CQ	–	NA	0.17	ND
Artemisinin (ng/mL)	–	NA	ND	0.15

*CQ* chloroquine, *CQ*^*S*^ chloroquine sensitive, *CQ*^*R*^ chloroquine resistant, *NA* not applicable, *ND* not determine

#### Transmission electron microscopy

Control and drug-treated parasites were washed with 1X PBS and suspended for 24 h with a mixture of a solution containing glutaraldehyde (2.5%) and paraformaldehyde (4%) in cacodylate buffer (0.1 M) having pH 7.2, followed by post-fixing with a mixture of osmium tetroxide (1% OsO_4_) and potassium ferrocyanide (0.8%) in cacodylate buffer (0.1 M). The pellet down cells were then dehydrated in a graded concentration of acetone and embedded in Epoxy resin (EPON mixture with DMP in rubber moulds). Further kept for polymerization at 60 °C for 24 h. Ultra-thin sections were cut on Ultracut-E ultramicrotome (Reichert-Jung, Germany) using a diamond knife and mounted on copper grids. The sections were double-stained with uranyl acetate and lead citrate and visualized in a ZEISS EM900 and in a JEOL 1200EX transmission electron microscope.

### Results and discussion

In a search for novel compounds that would be effective blood schizonticides against both CQ sensitive and resistant *P. falciparum*, the action of chalcones derivatives at an ultra-structural level under in vitro conditions was studied. Infected RBCs [CQ sensitive (MRC-2) and CQ resistant (RKL-9) *Plasmodium* strain] were incubated without drug, taken as negative control, and treated with three chalcone derivatives 1, 2 and 3 and standard CQ and artemisinin taken as positive control for CQ sensitive (MRC-2) and CQ resistant (RKL-9) strain at twice their respective IC_50_ values for 24 h and then harvested, washed, fixed, embedded and stained to visualize ultra-structure changes before and after intervention of treatment under in vitro condition on both the *Plasmodium* strain through transmission electron microscope. Released *Plasmodium* from lysed RBC has shown in (Additional file 1: Figure S1).

**Fig. 1:**
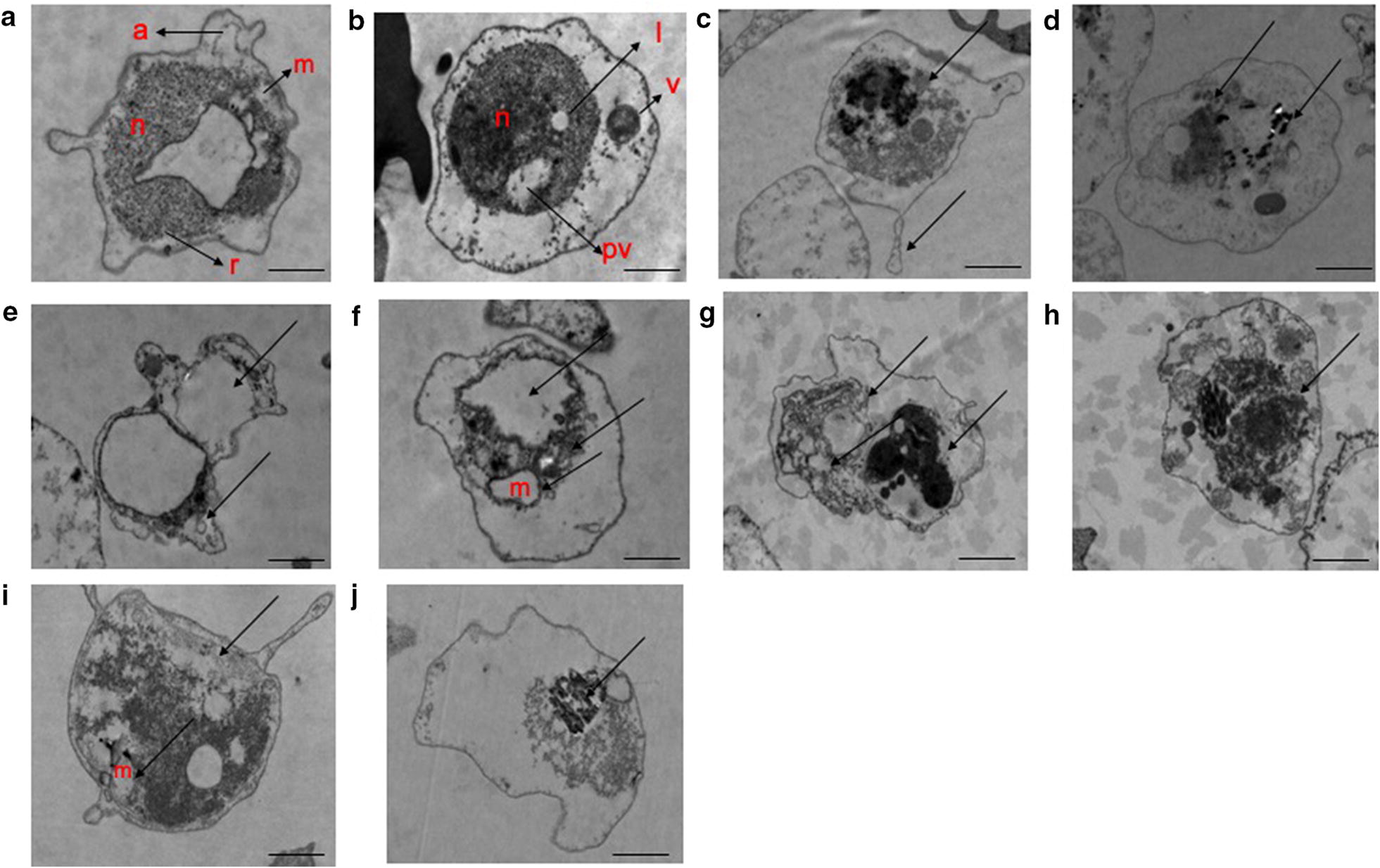
Electron micrographs depicting the effect of chalcone derivatives on *P. falciparum* CQ sensitive (MRC-2) strain. **a** untreated control “early ring stage (early trophozoite)” and **b** untreated control “late ring stage (late trophozoite)” **c**, **d** CQ-treated; **e**, **f** compound 1-treated; **g**, **h** compound 2-treated; **i**, **j** compound 3-treated. **a** Arrows represent appendages (a), ribosomes (r) and, mitochondrion (m). **b** Arrows represent nucleus (n), lipid vacuole (l), a cytostome with a forming food vacuole (v), and pigment vacuole (pv). **c**–**j** represents degenerative *Plasmodium* with condensed chromatin, vacuolation, membrane disintegration, swollen mitochondrion. The bar represents 800 nm-1 µm

At the ultrastructural level, the most conspicuous changes are seen in trophozoites at all stages of development. After 24 h of drug exposure, the mitochondria were swollen and there was the loss of membranes integrity. The endoplasmic reticulum is less conspicuous and ribosome numbers are markedly decreased (Fig. 1c–j) compared with parasites of the same stage from untreated control, having cytoplasm with well-distributed ribosomes and a clearly defined nucleus. However, food vacuole was well integrated on CQ treatment as compared to chalcones shown in (Fig. 1c, d).

**Fig. 2:**
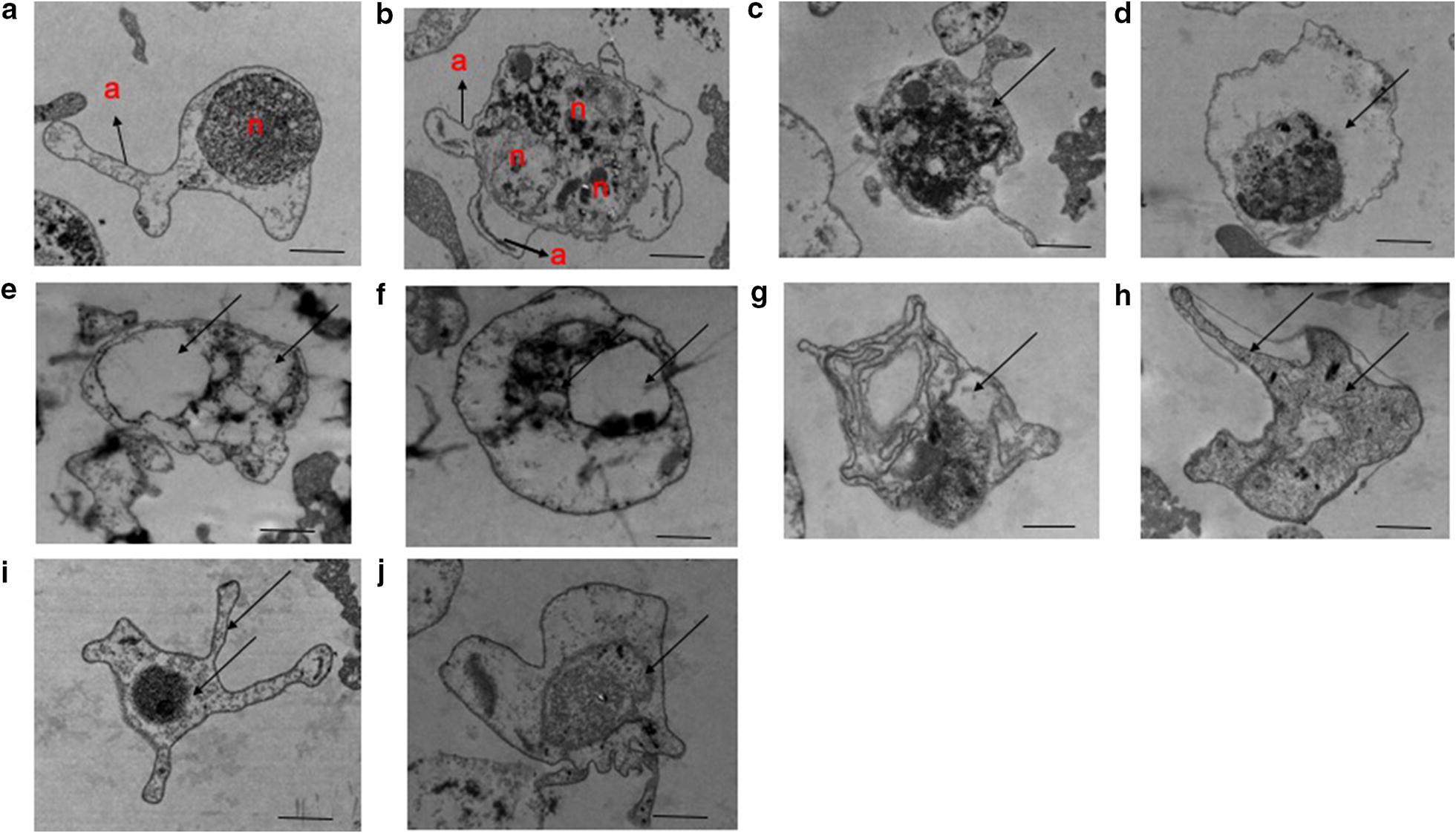
Electron micrographs depicting the effect of chalcone derivatives on *P. falciparum* CQ resistant (RKL-9) strain. **a** untreated control “early ring stage (early trophozoite)” and **b** Untreated Control “Developing Schizont”; **c**, **d** artemisinin-treated; **e**, **f** compound 1-treated; **g**, **h** compound 2-treated; **i**, **j** compound 3-treated. **a** Arrow indicates appendages, **b** arrows represent appendages (a) and multiple nuclei (n). **c**–**j** Arrows show disintegrated forms of the malaria parasite, observed through darkly stained food vacuole **c**, **d**, **f**, **i**, larger vacuole formation (**d**–**j**), disintegrated and disoriented parasite membranes and organelles, ribosome loss along with condensed nuclei (**c**–**j**). The bar represents 800 nm–1 µm

Although the parasites are advancing towards schizogony, this process is being blocked. In Fig. 2. RKL-9 (c–j), the nuclear membranes appear to have completely disappeared in places, making it difficult to distinguish between nuclear contents and the general cytoplasm. There is also a marked loss of endoplasmic reticulum and of ribosome density and content, especially in the parasite shown in Fig. 2. RKL-9 (d–j). In both types, mitochondria are distended, nuclear membranes blebbed, and ribosomes are reduced. Numerous ribosomes are still visible but irregularly distributed within the cytoplasm which lacks the endoplasmic reticulum. Swollen mitochondria with varying degree of membrane irregularities is seen which shows similar results as compared with earlier report done on Leishmania parasite under the effect of oxygenated chalcones licochalcone A [14] and 2,4-dimethoxy-4′-butoxy-chalcone [15]. The peripheral parasite membrane is also partially disrupted, as is the membrane of the pigment vesicles. A major feature was the appearance of large vacuoles (Figs. 1 and 2d–j). Schizogony has been interrupted. The process of merozoite membrane formation, both peripherally and around the nuclei, is clearly interrupted. The ultrastructural changes demonstrate the significant disturbance of all parasite membranes, including those of the nucleus, mitochondria, and endoplasmic reticulum, in association with a marked reduction of ribosomes in the trophozoites and developing schizonts which is supported by previous studies where drugs like artemisinin, dipyridamole, artesunate, quinine, and piperaquine showed more or less similar effects [16–18]. However, loss of knobs on drug treatment was not observed in both the *Plasmodium* strains due to RBC-free parasite. Knobs present on *P. falciparum* responsible for carrying antigens which benefit in parasite sequestration, would explain virulence and cytoadhesion ability [19]. Also, the food vacuole was intact but in condensed form in case of CQ-treated parasite, however, chalcone-treated parasites showed disintegrated food vacuole in mostly both CQ-sensitive and CQ-resistant strain. Additionally, evidence of darkly stained food vacuole in electron micrograph (Additional file 2: Figure S2) reveals interference in the haemoglobin degradation which ultimately lead to the death of the parasite [20]. Thus, suggest food vacuole may be the optimum target for chalcones which is further supported by our previous finding on significant reduction in hemozoin formation after chalcone treatment [11].

### Conclusion

Chalcones and their derivatives are important scaffolds for providing various potent leads. The present ultrastructure study indicates food vacuole as the chief target site of these chalcone derivatives. However, the disintegration of other organelles, chromatin condensation and a marked reduction in ribosomes provide evidence about multiple mechanisms by which these chalcones exert its antiparasitic effect and it needs to be further validated through various biochemical, molecular and proteomics tools. Also, there is no such published study concerning the ultrastructural alteration induced on chalcone or chalcone derivative treatment on malaria parasite. Henceforth, the present study opens up perspectives for further exploration of these derivatives in vivo malaria models to discover more about its effect and mechanism of action.

## Limitations

The stage dependent growth inhibition as well as time dependent growth inhibition of the parasite was not evaluated in the current study, which may provide clarity of kinetic and morphological changes inside the parasite under the influence of these chalcone derivatives.

## Supplementary information

**Additional file 1: Figure S1.** Electron micrographs of released *Plasmodium* from lysed RBC depicting various stages in the asexual cycle of *P. falciparum.* RBC: Red Blood Cells; n: nucleus; pv: pigment vacuole; v: a cytostome with a forming food vacuole; l: lipid vacuole; dm: developing merozoites. The bar represents 800 nm.

**Additional file 2: Figure S2.** Electron micrographs depicting the effect of treatment on hemozoin formation by malaria parasite compared to untreated control. A) untreated trophozoite, B) untreated developing schizont, C) & D) CQ-treated and E) & F) Compound-treated. Circle areas show haemoglobin degradation and formation of hemozoin crystals. CQ and Compound-treated, (C & E) show darkly stained food vacuole due to interference in haemoglobin digestion; (D & F) malaria parasites shows total disorganization with the residue of hemozoin crystals. The bar represents 600nm-1μm.

## Data Availability

All data generated or analyzed during this study are included in this published article and its additional information files.

## Abbreviations

CQ: Chloroquine
DMSO: Dimethyl sulfoxide
HEPES: 4-(2-hydroxyethyl)-1-piperazineethanesulfonic acid
IC_50_: The half maximal inhibitory concentration
NaHCO_3_: Sodium bicarbonate
PBS: Phosphate buffer saline
RBC: Red Blood Cell
