# Supplementary figures and images for: Ultrastructural alterations in Plasmodium falciparum induced by chalcone derivatives
Source: BMC Res Notes. 2020 Jun 15;13:290. doi: 10.1186/s13104-020-05132-z (PMC7296763; doi:10.1186/s13104-020-05132-z)

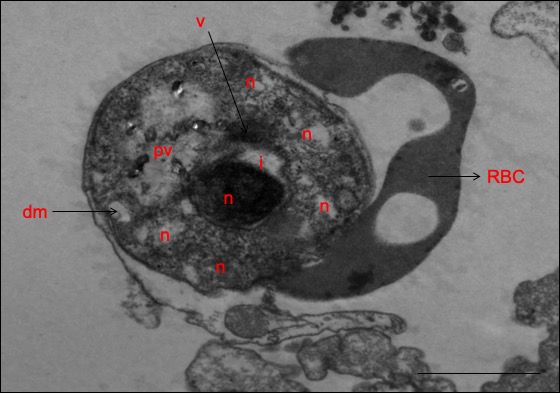

Supplement: Supplementary file 1 — Additional file 1: Figure S1. Electron micrographs of released Plasmodium from lysed RBC depicting various stages in the asexual cycle of P. falciparum. RBC: Red Blood Cells; n: nucleus; pv: pigment vacuole; v: a cytostome with a forming food vacuole; l: lipid vacuole; dm: developing merozoites. The bar represents 800 nm. [file 13104_2020_5132_MOESM1_ESM.jpg]

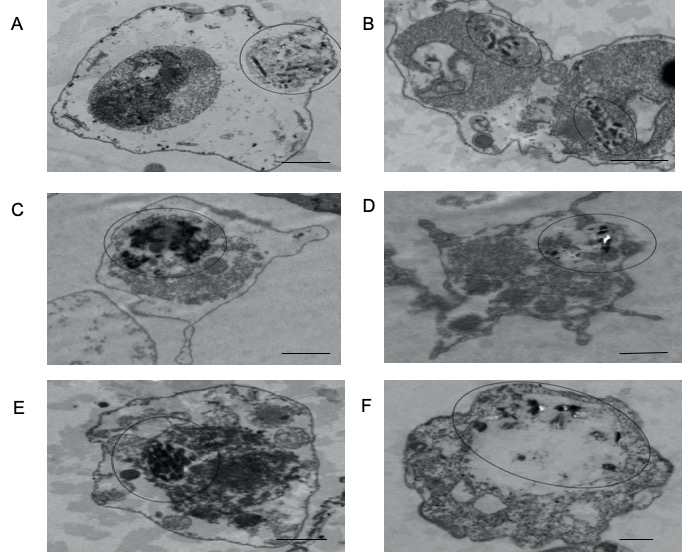

Supplement: Supplementary file 2 — Additional file 2: Figure S2. Electron micrographs depicting the effect of treatment on hemozoin formation by malaria parasite compared to untreated control. A) untreated trophozoite, B) untreated developing schizont, C) & D) CQ-treated and E) & F) Compound-treated. Circle areas show haemoglobin degradation and formation of hemozoin crystals. CQ and Compound-treated, (C & E) show darkly stained food vacuole due to interference in haemoglobin digestion; (D & F) malaria parasites shows total disorganization with the residue of hemozoin crystals. The bar represents 600nm-1μm. [file 13104_2020_5132_MOESM2_ESM.jpg]
